# Carbonic anhydrase 2‐like in the giant clam, *Tridacna squamosa*: characterization, localization, response to light, and possible role in the transport of inorganic carbon from the host to its symbionts

**DOI:** 10.14814/phy2.13494

**Published:** 2017-12-04

**Authors:** Yuen K. Ip, Clarissa Z. Y. Koh, Kum C. Hiong, Celine Y. L. Choo, Mel V. Boo, Wai P. Wong, Mei L. Neo, Shit F. Chew

**Affiliations:** ^1^ Department of Biological Sciences National University of Singapore Singapore; ^2^ The Tropical Marine Science Institute National University of Singapore Singapore; ^3^ St. John's Island National Marine Laboratory National University of Singapore Singapore; ^4^ Natural Sciences and Science Education National Institute of Education Nanyang Technological University Singapore

**Keywords:** Bicarbonate, calcification, carbon dioxide, shell formation, *Symbiodinium*, zooxanthellae

## Abstract

The fluted giant clam, *Tridacna squamosa*, lives in symbiosis with zooxanthellae which reside extracellularly inside a tubular system. Zooxanthellae fix inorganic carbon (C_i_) during insolation and donate photosynthate to the host. Carbonic anhydrases catalyze the interconversion of CO_2_ and ${\text{HCO}}_{3}^{-}$, of which carbonic anhydrase 2 (CA2) is the most ubiquitous and involved in many biological processes. This study aimed to clone a *CA2* homolog (*CA2‐like*) from the fleshy and colorful outer mantle as well as the thin and whitish inner mantle of *T. squamosa*, to determine its cellular and subcellular localization, and to examine the effects of light exposure on its gene and protein expression levels. The cDNA coding sequence of *CA2‐like* from *T. squamosa* comprised 789 bp, encoding 263 amino acids with an estimated molecular mass of 29.6 kDa. A phenogramic analysis of the deduced CA2‐like sequence denoted an animal origin. CA2‐like was not detectable in the shell‐facing epithelium of the inner mantle adjacent to the extrapallial fluid. Hence, CA2‐like is unlikely to participate directly in light‐enhanced calcification. By contrast, the outer mantle, which contains the highest density of tertiary tubules and zooxanthellae, displayed high level of *CA2‐like* expression, and CA2‐like was localized to the tubule epithelial cells. More importantly, exposure to light induced significant increases in the protein abundance of CA2‐like in the outer mantle. Hence, CA2‐like could probably take part in the increased supply of inorganic carbon (C_i_) from the host clam to the symbiotic zooxanthellae when the latter conduct photosynthesis to fix C_i_ during light exposure.

## Introduction

The fluted giant clam, *Tridacna squamosa*, belongs to Phylum: Mollusca, Class: Bivalvia, Order: Veneroida, Family: Cardiidae, Subfamily: Tridacninae. It inhabits the shallow tropical water of the Indo‐Pacific coral reefs (Klumpp et al. 1992; Neo et al. 2017). Unlike the cold and nutrient‐rich waters of higher latitudes, the warm tropical waters lack overturn which stirs up bottom sediments and brings nutrients to the surface waters to trigger plankton growth (Horn 2003). Hence, tropical waters are usually low in plankton content and nutrient deficient (De Goeij et al. 2013). Despite the nutrient‐poor environment, giant clams in tropical waters can maintain high growth rates (Rosewater 1965) because they live in symbiosis with single‐celled microalgae (also known as zooxanthellae) of genus *Symbiodinium* (Clades A, C, and D) (Trench 1987; Hernawan 2008).

Most symbiotic marine invertebrates, like hard corals and sea anemones, harbor zooxanthellae intracellularly within symbiosomes, but in giant clams, the symbiotic zooxanthellae reside extracellularly inside a tubular system (Norton and Jones 1992; Norton et al. 1992). This tubular system originates from the clam's stomach, and splits into smaller secondary and tertiary tubules that permeate the fleshy outer mantle which can be extended beyond the edge of the shell valves when exposed to sunlight (Norton et al. 1992; Yellowlees et al. 1993). The extensible outer mantle has distinctive coloration (Todd et al. 2009) due to the presence of high densities of symbiotic zooxanthellae and host iridophores which focus light of the appropriate wavelength for photosynthesis to the symbionts (Griffiths et al. 1992). With insolation, the symbionts undergo photosynthesis and share with the host the photosynthate (Muscatine 1990), which can constitute up to 100% of the host's energy requirements (Klumpp et al. 1992). In return, the host supplies essential nutrients like inorganic carbon (C_i_), nitrogen, and phosphorus to the symbionts in support of their metabolism and growth (Furla et al. 2005).

Shell formation in giant clams involves the precipitation of calcium carbonate (CaCO_3_), according to the equation Ca^2+^ + ${\text{HCO}}_{3}^{-}$ ⇌ CaCO_3_ + H^+^, from the extrapallial fluid onto the inner surfaces of the shell valves. The calcification process requires the participation of the thin and mostly nonpigmented inner mantle tissue delineated by the shell's pallial line. The inner mantle secretes Ca^2+^, ${\text{HCO}}_{3}^{-}$, and an organic matrix to the extrapallial fluid where calcification occurs, and CaCO_3_ is deposited onto the inner surface of the shell valve (Wilbur and Watabe 2006). Light‐enhanced calcification refers to the unique phenomenon of calcification occurring at a faster rate in light than in darkness (Chalker and Taylor 1975), which was first observed in scleractinian corals (Kawaguti and Sakumoto 1948). There is evidence indicating that light‐enhanced calcification also occurs in giant clams (Sano et al. 2012), whose growth rates are critically dependent on the availability of light (Crawford et al. 1987).

Taken together, two processes are accelerated when giant clams are exposed to light. First, the symbiotic zooxanthellae photosynthesize at a high rate. Second, the rate of shell formation increases due to light‐enhanced calcification. As both processes require C_i_ as substrates, there is a high demand for C_i_ by the giant clam–zooxanthellae association during insolation. In fact, photosynthetic CO_2_ fixation by zooxanthellae can completely deplete C_i_ in the clam hemolymph in <13 min if not replenished by either endogenous respiratory CO_2_ or exogenous C_i_ from seawater (Rees et al. 1993), during which, the two processes of photosynthesis in the symbionts and calcification in the host would be competing for the endogenous pool of C_i_.

In aqueous solution, C_i_ is present mainly as dissolved CO_2_ and ${\text{HCO}}_{3}^{-}$, which can interconvert according to the equation CO_2_ + H_2_O ⇌ H_2_CO_3_ ⇌ ${\text{HCO}}_{3}^{-}$ + H^+^. Even in the absence of a catalyst, the hydration of CO_2_ proceeds at a moderate pace, with an effective first‐order rate constant of 0.15 sec^−1^ in water, while the dehydration of H_2_CO_3_ is relatively rapid, with a first‐order rate constant of 50 sec^−1^ (Maren 1967). These rate constants correspond to an equilibrium constant of *K*_1_ = 5.4 × 10^−5^ and a ratio of 340:1 for [CO_2_] to [H_2_CO_3_]. Notwithstanding the moderate uncatalyzed rates of CO_2_ hydration and H_2_CO_3_ dehydration, almost all organisms possess carbonate anhydrases (CAs; EC 4.2.1.1) which are zinc‐containing enzymes catalyzing these reactions with dramatic acceleration of CO_2_ hydration (Supuran 2008b). CAs are needed because CO_2_ hydration and ${\text{HCO}}_{3}^{-}$ dehydration are commonly coupled to rapid physiological and biochemical processes, particularly in relation to transport phenomena. CAs comprise four genetically distinct families (*α*,* β*,* γ*, and *δ*), with *α*‐CA being the largest and the most ubiquitous family (Chegwidden et al. 2000). In humans, 15 *α*‐CA isoforms with varying subcellular localizations, tissue distribution, and enzyme kinetics have been identified (Aggarwal et al. 2013). They play vital roles in a wide range of physiological processes involving CO_2_/${\text{HCO}}_{3}^{-}$, such as respiration, acid/base homeostasis, electrolyte secretion, bone resorption, and calcification (Supuran 2008b). Among all the CA isoforms, CA2 has the highest catalytic activity, rapidly catalyzing the hydration of CO_2_ to ${\text{HCO}}_{3}^{-}$ with a turnover in the order of 10^6^ molecules sec^−1^ (Purkerson and Schwartz 2007). It is almost universally expressed in major mammalian tissues, and is the primary CA found in red blood cells, osteoclasts, and renal tubules (Chegwidden et al. 2000).

In giant clams, CAs have been proposed to take part in the acquisition of C_i_ and the facilitation of C_i_ movement between tissues and organs (Leggat et al. 2002; Yellowlees et al. 2008). Specifically, two distinct CA isoforms with molecular masses of 32 kDa and 70 kDa have been detected in the ctenidium (or gill) and mantle of the true giant clam, *Tridacna gigas* (Baillie and Yellowlees 1998; Leggat et al. 2002, 2005). However, no molecular information on CA2 or its homolog is available for giant clams in general. Therefore, this study was undertaken to clone, sequence, and characterize a *CA2* homolog (*CA2‐like*) from the outer and inner mantle of *T. squamosa*. As described previously (Hiong et al. 2017b), the portion of the thin and whitish mantle demarcated by the pallial line on the inside surface of the shell valve is regarded as the inner mantle, while the fleshy, colorful, and extensible portion of the mantle outside the pallial line is regarded as the outer mantle. Particularly, the thin inner mantle comprises a shell‐facing epithelium and a seawater‐facing epithelium separated by loose connective tissues and hemolymph. After obtaining the complete coding sequence of *CA2‐like*, its gene expression levels in various tissues and organs were examined through polymerase chain reaction (PCR). Furthermore, the effects of 3, 6, or 12 h of light exposure on the mRNA expression levels of *CA2‐like* in the outer and inner mantle of *T. squamosa*, as compared with the control kept in darkness for 12 h, were determined. Based on the deduced CA2‐like sequence, an anti‐CA2‐like antibody was custom‐made to examine the effects of light exposure on its protein abundance in the outer and inner mantle of *T. squamosa*. It was hypothesized that the gene and protein expression levels of *CA2‐like*/CA2‐like in the outer and inner mantle, like those of Na^+^/H^+^ exchanger 3‐like transporter (Hiong et al. 2017b) and glutamine synthetase (Hiong et al. 2017a) in the ctenidium, would be upregulated by light exposure. In order to shed light on the possible physiological function of CA2‐like in *T. squamosa*, immunofluorescence microscopy was also performed to confirm its cellular localization. The assumption was that CA2‐like would be localized to the epithelial cells of the tubules surrounding the zooxanthellae if it took part in the delivery of C_i_ to the symbionts, but it would be localized to the shell‐facing epithelium of the inner mantle if it participated directly in light‐enhanced calcification.

## Materials and Methods

### Human and animal rights

No institutional (National University of Singapore Institutional Animal Care and Use Committee) approval is required for invertebrates including giant clams at the time the laboratory experiments were performed. The animals were anesthetized with 0.2% phenoxyethanol before killing to minimize any stress and suffering.

### Experimental animals

Specimens of *T. squamosa* (521 ± 184 g; *N *=* *24) were obtained from Xanh Tuoi Tropical Fish Co., Ltd. (Vietnam), and kept in an indoor aquarium. They were maintained in 350 L of recirculating seawater in a glass tank (L 90 cm × W 62 cm × H 60 cm) under a 12‐h light:12‐h dark regime, with seawater conditions and light intensity as described previously (Ip et al. 2015), except that the seawater temperature was maintained at 26 ± 1°C.

### Experimental conditions and tissue collection

For molecular work, four individuals of *T. squamosa* (*N *=* *4; control) were sampled at the end of the 12‐h dark period of the 12‐h light:12‐h dark regime. In addition, four individuals (*N *=* *4 for each time point) were sampled each at 3, 6, or 12 h after exposure to light (a total of 12 individuals). Parallel controls were not used in order to accurately simulate the dark/light hours experienced by the clams in their natural habitat. After being anesthetized in 0.2% phenoxyethanol, the giant clam was forced open, and the adductor muscle was cut. The pigmented outer mantle was dissected, and the nonpigmented inner mantle was excised. Samples of the ctenidia, hepatopancreas, foot muscle, byssal retractor muscle, adductor muscle, kidney, and heart were also collected. Collected tissue samples were blotted dry, immediately freeze clamped with aluminum tongs precooled by liquid nitrogen, and stored at −80°C until analysis. Tissues for immunofluorescence microscopy were sampled from *T. squamosa* which had been exposed to darkness or light for 12 h (*N *=* *4 each) and anesthetized in 0.2% phenoxyethanol.

### Total RNA extraction and cDNA synthesis

The extraction and purification of total RNA from the tissues of *T. squamosa* were performed using TRI Reagent™ (Sigma‐Aldrich, St. Louis, MO) and RNeasy Plus Mini Kit (Qiagen, Hilden, Germany), respectively. The purified RNA was quantified by a Shimadzu BioSpec‐nano spectrophotometer (Shimadzu, Tokyo, Japan), and its integrity was determined by electrophoresis. Then cDNA was synthesized from 4 *μ*g of purified RNA using a RevertAid™ first‐strand cDNA synthesis kit (Thermo Fisher Scientific, Waltham, MA).

### PCR, cloning, and RACE‐PCR

In order to obtain the partial *CA2‐like* sequence from the outer and inner mantle of *T. squamosa*, degenerate primers (forward: 5′‐GGGWTACGAKRAKCATAATGG‐3′ and reverse: 5′‐CCCAGRTAGGTCCAGTAKTY‐3′) were designed based on the conserved regions of *Crassostrea gigas CA2* (XM_011449590.2), *Mytilus galloprovincialis CA2* (KT818923.1), *Octopus bimaculoides CA2‐like* (XM_014929272.1), and *Danio rerio CA2* (NM_199215.1). PCR and cloning experiments were performed as described previously in Hiong et al. (2017a). Analysis of multiple clones of *CA2‐like* fragments from the outer and inner mantle of *T. squamosa* revealed only one form. In order to obtain the complete coding sequence of *CA2‐like*, 5′ and 3′ RACE (SMARTer RACE cDNA amplification kit; Clontech Laboratories, Mountain View, CA) were performed using the following primers: 5′‐ACCTGTGTTCGCAATTTCAAGACCTGAC‐3′ and 5′‐GGATTATCGCCAATATCAGACGGAGCCC‐3′.

Samples were processed for sequencing using BigDye Terminator v3.1 Cycle Sequencing Kit (Thermo Fisher Scientific) with subsequent ethanol/sodium acetate precipitation. Purified products were automatically sequenced using the 3130XL Genetic Analyzer (Thermo Fisher Scientific). Sequence data were analyzed with BioEdit version 7.2.5 (Hall 1999). The cDNA sequence of *CA2‐like* has been deposited into GenBank (accession number MF042362).

### Gene expression in various organs/tissues

The gene expression of *CA2‐like* in the cDNAs of outer mantle, inner mantle, ctenidia, hepatopancreas, foot muscle, byssal retractor muscle, adductor muscle, kidney, and heart of *T. squamosa* by PCR using a set of gene‐specific primers (forward: 5′‐TCCATTACAACGAGAAGTACGG‐3′ and reverse: 5′‐GGCGATAATCCATCATACTGCT‐3′). Each PCR was performed in 10 *μ*l reaction volume with thermal cycling conditions of 94°C for 3 min, followed by 24 cycles of 94°C for 30 sec, 50°C for 30 sec, 72°C for 1 min, and 1 cycle of final extension at 72°C for 10 min. The products were then separated on a 1% agarose gel by gel electrophoresis.

### Deduced amino acid sequence and phenogramic analyses

The *CA2‐like* sequence obtained from *T. squamosa* was translated using the ExPASy Proteomic server (http://web.expasy.org/translate/) (Gasteiger et al., 2003) to obtain the deduced CA2‐like amino acid sequence. In order to verify its identity and host origin, the CA2‐like sequence from *T. squamosa* was aligned and compared with various CA sequences from humans and algae, and *T. gigas* CA using BioEdit (Hall 1999). Selected CA sequences from GenBank were then used to conduct phenogramic analyses via the neighbor‐joining method with 100 bootstrap replicates using Phylip programs (Felsentein 1989).

### Quantitative real‐time PCR

RNA (4 *μ*g) was extracted from the inner and outer mantle of *T. squamosa* as mentioned above and reverse‐transcribed using random hexamer primers with RevertAid™ first strand cDNA synthesis kit. qPCR was performed in triplicates using a StepOnePlus™ Real‐Time PCR System (Applied Biosystems). The mRNA expression level of *CA2‐like* was determined using specific forward (5′‐TCCATTACAACGAGAAGTACGG‐3′) and reverse (5′‐GGCGATAATCCATCATACTGCT‐3′) qPCR primers. The qPCR reactions contained 5 *μ*L of 2X Fast SYBR^®^ Green Master Mix (Applied Biosystems), 0.3 *μ*mol L^−1^ of forward and reverse primers each, and various quantities of standard (to construct the standard curve) or 1 ng of sample cDNA in a total volume of 10 *μ*L. The cycling conditions, melt curve analysis, and construction of a standard curve were performed according to the method of Hiong et al. (2017a,b). The amplification efficiency of the *CA2‐like* primers was 97.4%. The quantity of *CA2‐like* transcripts in a sample was determined from the linear regression line derived from the standard curve and expressed as copy number per ng of total RNA.

### SDS‐PAGE and western blotting

A custom‐made anti‐CA2‐like polyclonal antibody (epitope sequence PLTKDDWGYEDHNG) was raised in rabbits by a commercial firm (GenScript, Piscataway, NJ). Protein extraction and SDS‐PAGE were performed according to the methods of Hiong et al. (2017a,b). Fifty micrograms of protein were electrophoretically separated and transferred onto PVDF membranes. Membranes were then blocked with 5% skim milk in TTBS (pH 7.6) for 1 h at 25°C, and incubated with anti‐CA2 antibodies (1.25 *μ*g mL^−1^) or anti‐*α*‐tubulin antibodies (12G10, 1.25 *μ*g mL^−1^) for 1 h at 25°C. The primary antibodies were diluted in TTBS prior to use. Subsequently, the membranes were incubated in alkaline phosphatase‐conjugated secondary antibodies (Santa Cruz Biotechnology, Inc.; 0.04 *μ*g mL^−1^) for 1 h at 25°C. Bands were visualized using a BCIP/NBT Substrate Kit (Life Technologies). The blots were scanned using a CanoScan 9000F Mark II flatbed scanner in TIFF format at 600 dpi resolution. The optical density of the band was quantified using ImageJ (version 1.50, NIH), calibrated with a 37‐step reflection scanner scale (1″ × 8″; Stouffer #R3705‐1C). Results were presented as relative protein abundance of CA2‐like normalized with *α*‐tubulin. In order to validate the specificity of the anti‐CA2‐like antibody, the primary antibody (25 *μ*g) was incubated with the immunizing peptide (125 *μ*g) for 1 h at 25°C. The incubated mixture was diluted in TTBS and the aforementioned western blotting procedure was carried out as usual.

### Immunofluorescence microscopy

Samples of outer mantle and inner mantle were excised and immersed in 3% paraformaldehyde diluted in seawater at 4°C overnight. Sample preparation and immunostaining were performed following the method of Hiong et al. (2017b) except that the antigen retrieval was carried out using 0.05% citraconic anhydride with heating at 90°C for 10 min, and the concentration of the custom‐made anti‐CA2 rabbit polyclonal antibody (Genscript) used was 1.67 *μ*g mL^−1^.

After immunostaining, sections were viewed under an Olympus BX60 epifluorescence microscope (Olympus Corporation, Tokyo, Japan) mounted with an Olympus DP73 digital camera (Olympus Corporation) for image capturing. All images were captured under predetermined optimal exposure settings. Of note, zooxanthellae are known to contain pigments that autofluoresce (Castillo‐Medina et al. 2011), therefore, they were visualized using a U‐MWIG filter (Olympus) with excitation at 520‐550 nm and 580 nm band‐pass emission filter (red channel). CA2‐like immunostaining was observed using the U‐MNIBA filter (Olympus) with excitation at 470–490 nm and 515–550 nm band‐pass emission filter (green channel). Corresponding differential interference contrast (DIC) images were captured for tissue orientation.

### Statistical analysis

Results were presented as means ± SEM. SPSS version 21 (IBM Corporation, Armonk, NY) was used to perform statistical analyses. Levene's test was used to check homogeneity of variance. One‐way analyses of variance (ANOVA) followed by multiple comparisons of means by Dunnett's T3 (for unequal variance) or by Tukey's test (for equal variance) were used to evaluate differences among means and differences with *P *<* *0.05 were regarded as statistically significant.

## Results

### Nucleotide sequences, translated amino acid sequences, and phenogramic analysis

The complete cDNA coding sequence (789 bp) of *CA2‐like* was obtained from the outer and inner mantle of *T. squamosa* (GenBank accession number MF042362). No nucleotide polymorphism was observed between the *CA2‐like* cDNA sequences obtained from these two tissues. The open reading frame encoded for a 263‐amino acid polypeptide with an estimated molecular mass of 29.6 kDa.

**Figure 1:**
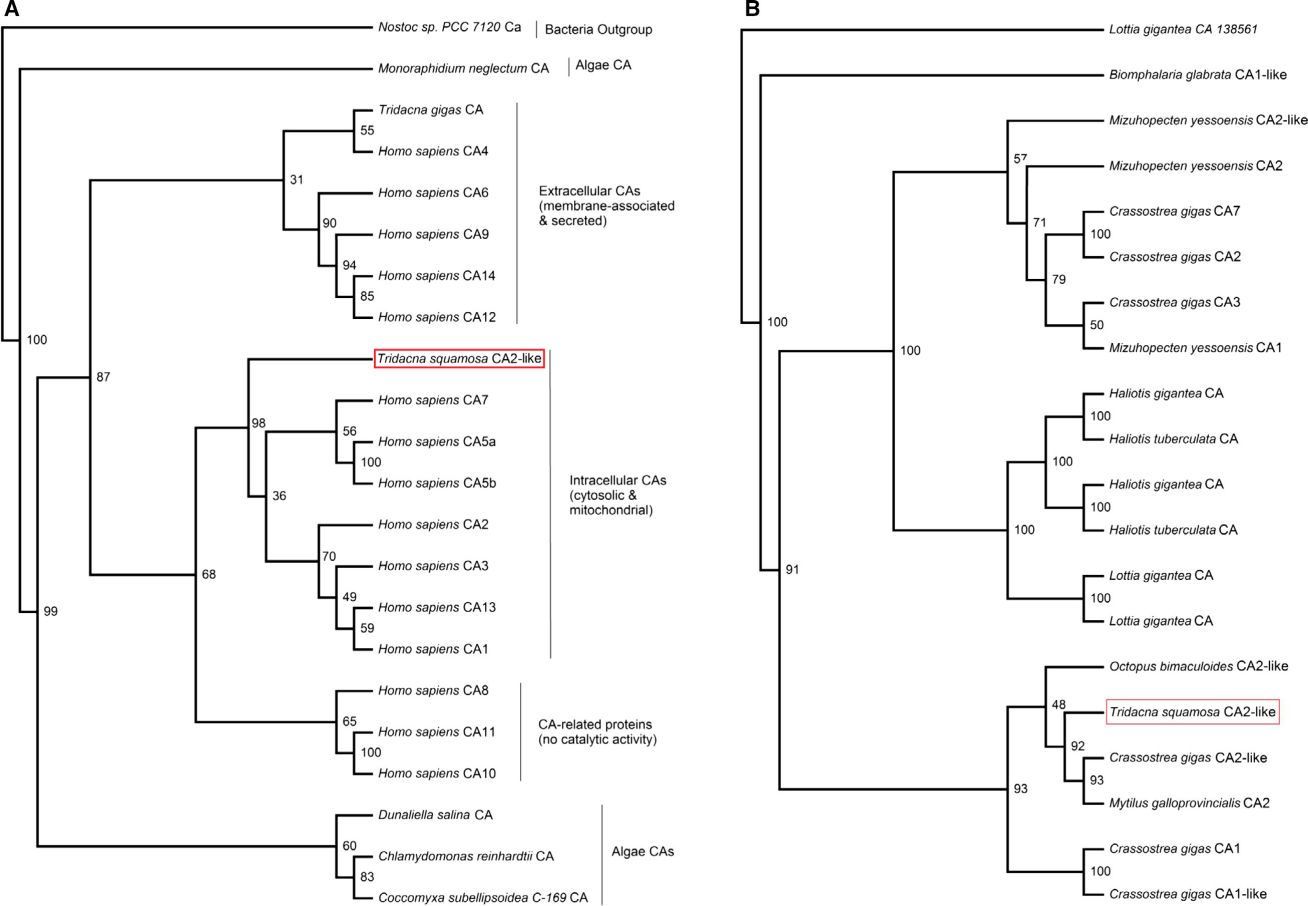
Phenogramic analyses of carbonic anhydrase 2‐like (CA2‐like) of *Tridacna squamosa*. (A) A phenogramic analysis of CA2‐like of *T. squamosa*, with all known CAs of *Homo sapiens*,* Tridacna gigas* CA (tgCA), and CAs of several species of algae. The Ca of the bacteria *Nostoc sp*. (*PCC7120*) was used as the outgroup. (B) A phenogramic analysis of CA2‐like of *T. squamosa*, with selected CAs of mollusks. The number located at each branch point represents the bootstrap value (max = 100).

The length of the deduced amino acid sequence of CA2‐like from *T. squamosa* was comparable to those of CA2 from other animal species. Phenogramic analyses showed that the CA2‐like of *T. squamosa* was grouped closely with the intracellular (cytosolic and mitochondrial) forms of CAs of *Homo sapiens*, distinctly separated from the CAs of algae (Fig. 1A), and also closely with CA2‐like of other species of mollusks (Fig. 1B). Furthermore, a sequence similarity analysis revealed that the CA2‐like of *T. squamosa* shared the highest similarity with the CA2 of another bivalve mollusk, *M. galloprovincialis* (60.9%; Table 1), but with the lowest similarity with CAs of algae (29.5–40.2%; Table 1), confirming its host (or clam) origin.

**Table 1:** Percentage similarities between the deduced amino acid sequence of *Tridacna squamosa* carbonic anhydrase 2‐like (CA2‐like; MF042362) and intracellular CA sequences from other species obtained from GenBank (accession numbers in brackets). Sequences are arranged in a descending order of similarity

Classification	Species	Similarity (%) with CA2‐like of *T. squamosa*
Molluscs	*Mytilus galloprovincialis* CA2 (ALF62133.1)	60.9
	*Crassostrea gigas* CA2 (EKC31880.1)	53.2
	*C. gigas* CA3 (EKC41232.1)	43.7
	*Tridacna gigas* CA (AAX16122.1)	24.8
Mammals	*Homo sapiens* CA2 (NP_000058.1)	61.8
	*Macaca fascicularis* CA2 (NP_001270614.1)	61.8
	*Bos taurus* CA2 (NP_848667.1)	58.8
	*B. taurus* CA1 (NP_001068934.1)	58.3
	*H. sapiens* CA3 (NP_005172.1)	57.2
	*B. taurus* CA5b (NP_001074377.2)	48.3
	*Mus musculus* CA5a (EDL11669.1)	36.1
Teleosts	*Danio rerio* Ca2 (NP_954685.1)	61.6
	*Oncorhynchus mykiss* Ca1 (NP_001117692.1)	60.1
	*D. rerio* Ca5a (NP_001104671.1)	50.0
	*Salmo salar* Ca5b (ACN10842.1)	48.3
Birds	*Gallus gallus* CA2 (NP_990648.1)	60.9
	*Ficedula albicollis* CA2 (XP_005042324.1)	59.5
	*Amazona aestiva* CA5a (KQK80107.1)	50.0
	*Manacus vitellinus* CA3 (KFW85838.1)	52.2
Amphibians	*Xenopus laevis* Ca2 (NP_001080080.1)	59.4
	*Xenopus tropicalis* Ca3 (NP_002939197.1)	55.5
	*Rana catesbeiana* Ca1 (ACO51997.1)	46.8
	*X. tropicalis* Ca5a (NP_001039155.1)	46.0
Arthropods	*Caligus rogercresseyi* CA2 (ACO11699.1)	59.4
	*Acyrthosiphon pisum* CA2 (NP_001280387.1)	56.5
	*Camponotus floridanus* CA1 (EFN65691.1)	41.4
	*Zootermopsis nevadensis* CA3 (KDR21152.1)	41.5
Algae	*Tetraselmis* sp*. GSL018* CA (JAC82403.1)	40.2
	*Chlamydomonas reinhardtii* CA (BAA14232.2)	34.4
	*Coccomyxa subellipsoidea C‐169* CA (EIE19267.1)	32.8
	*Dunaliella salina* CA (AAO83593.1)	29.5

**Figure 2:**
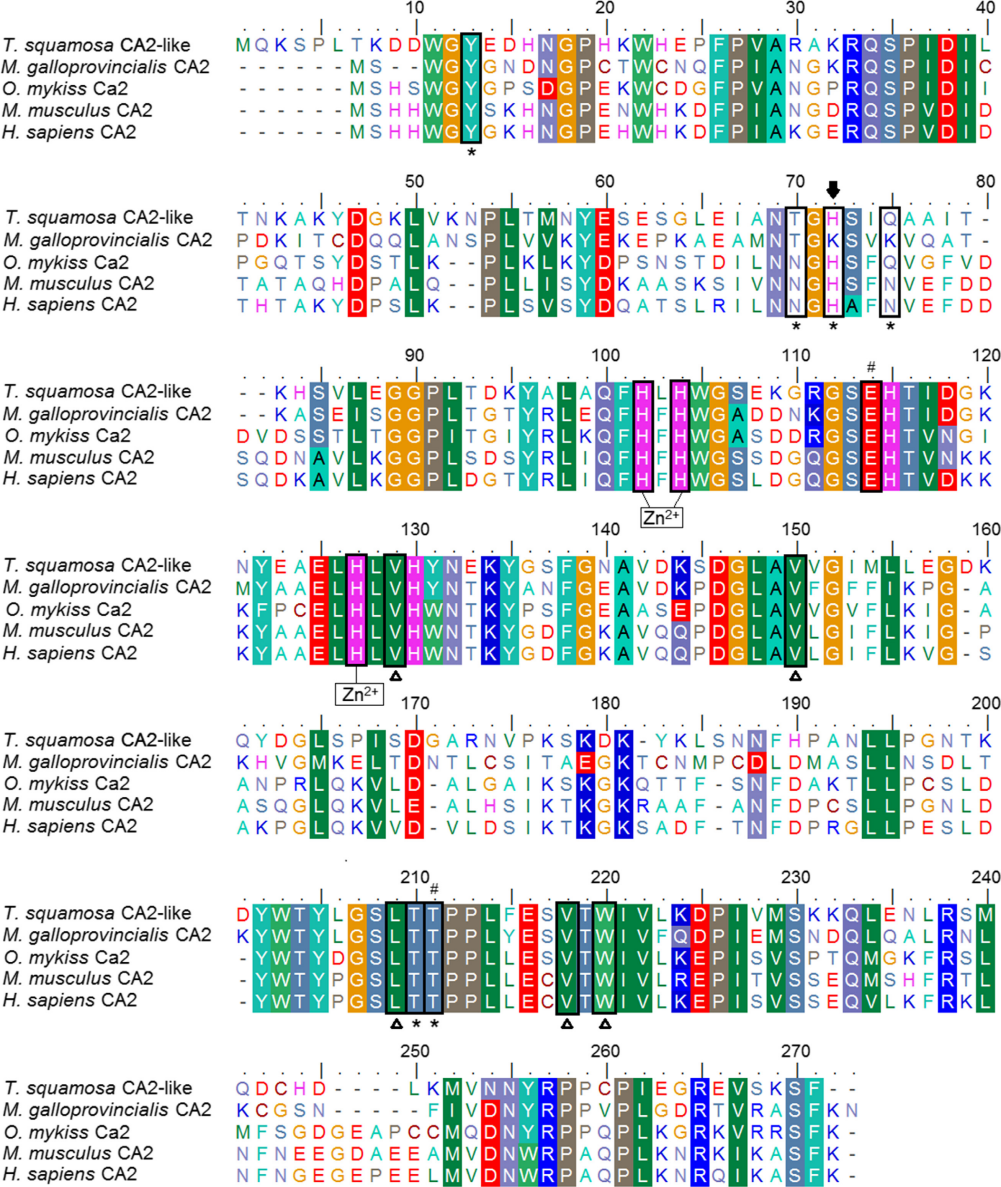
A multiple amino acid sequence alignment of carbonic anhydrase 2‐like (CA2‐like) of *Tridacna squamosa* with CA2 sequences from selected invertebrates and vertebrates. The shaded residues indicate identical or highly similar amino acids. The residues that coordinate the catalytic Zn^2+^ ion are labeled accordingly. Asterisks denote residues which form the hydrophilic portion of the CA2 active site. Open triangles indicate the residues that form the hydrophobic portion of the active site which binds CO_2_. Hash sign marks the gatekeeping residues. The amino acid residue that allows efficient proton‐transfer pathways is indicated by a block arrow.

An alignment of the deduced amino acid sequence of the CA2‐like of *T. squamosa* with those of human, mouse, fish, and mussel revealed that the residues essential for CA2 catalytic activity were highly conserved across these species (Fig. 2). Three conserved histidine residues (His102, His104, and His127; residues numbered according to CA2‐like of *T. squamosa* from the alignment in Fig. 2), which work with a highly polarized water/hydroxyl molecule to jointly coordinate the Zn^2+^ ion within its active site, were present in the CA2‐like of *T. squamosa*. The Zn^2+^ ion is known to be essential for the catalytic activity of all *α*‐CAs. The five residues that are specific to the hydrophobic portion of the CA2 active site (Val129, Val150, Leu209, Val218, and Trp220) and responsible for binding the substrate CO_2_ were fully conserved in the CAs of all the species analyzed (Fig. 2). The His64 proton‐shuttle residue of CA2 may contribute to its high CO_2_ hydration efficiency in *H. sapiens*, and this residue (His72) was conserved in the CA2‐like of *T. squamosa*. The gatekeeping residues Glu114 and Thr211 were also conserved (Fig. 2). However, the CA2‐like of *T. squamosa* differed from the CA2 of *H. sapiens* in two of the six amino acids which form the hydrophilic portion of its active site involved in proton transfer reactions (Asn70 → Thr70; Asn75 → Gln75). In both cases, the substituting residue was identical to either CA2 of mussel or teleost at the same location.

### mRNA expression of *CA2‐like* in various organs and tissues

**Figure 3:**
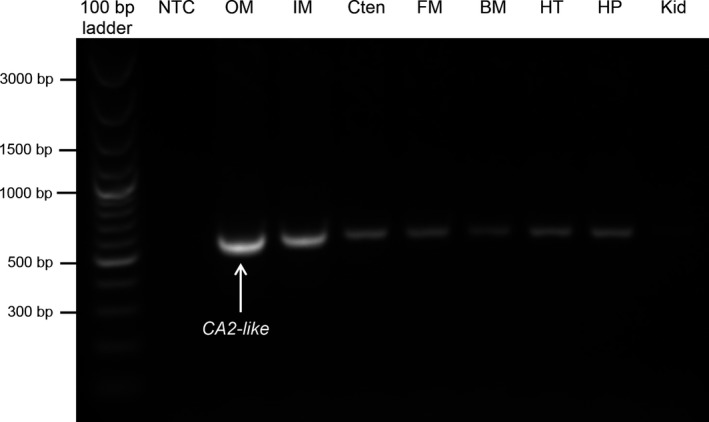
Gene expression of the *carbonic anhydrase 2‐like* (*CA2‐like*) in tissues/organs of *Tridacna squamosa*. The mRNA expression of *CA2‐like* in the outer mantle (OM), inner mantle (IM), ctenidium (Cten), foot muscle (FM), byssal muscle (BM), heart (HT), hepatopancreas (HP), and kidney (Kid) of *T. squamosa* kept in darkness for 12 h (control). A negative control (NTC) was included in the first lane.

The mRNA expression of *CA2‐like* was detected in all the organs/tissues examined, except the kidney, with particularly strong expressions in the outer and inner mantle (Fig. 3).

### Effects of light exposure on the mRNA expression levels of *CA2‐like*

**Figure 4:**
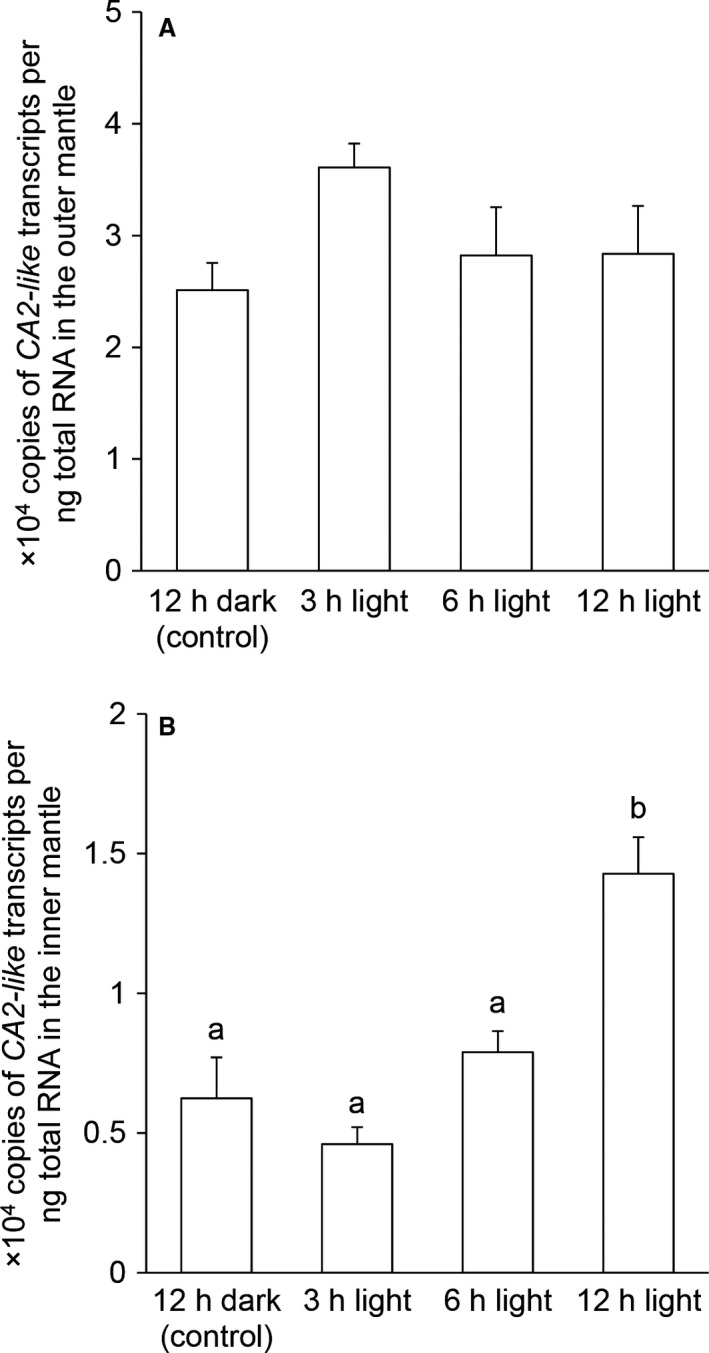
Effects of light on the mRNA expression levels of *carbonic anhydrase 2‐like* (*CA2‐like*) in the outer mantle and inner mantle of *Tridacna squamosa*. The transcript level (×10^4^ copies of transcripts per ng of total RNA) of the host *CA2‐like* in (A) the outer mantle and (B) the inner mantle of *T. squamosa* kept in darkness for 12 h (control), or exposed to light for 3, 6, or 12 h. Results represent means ± SEM (*N *=* *4). Means not sharing the same letters are significantly different from each other (*P < *0.05).

For *T. squamosa* kept in darkness for 12 h (control), the outer mantle, which contains the greatest density of zooxanthellae, had higher mRNA expression levels of *CA2‐like* (Fig. 4A) compared to the inner mantle (Fig. 4B). The transcript level of *CA2‐like* remained statistically unchanged in the outer mantle of *T. squamosa* after 3, 6, or 12 h of exposure to light as compared to the control kept in darkness (Fig. 4A). By contrast, there was a significant increase (~2.3‐fold) in the transcript level of *CA2‐like* in the inner mantle of clams exposed to light for 12 h (Fig. 4B).

### Effects of light exposure on the protein abundance of CA2‐like

**Figure 5:**
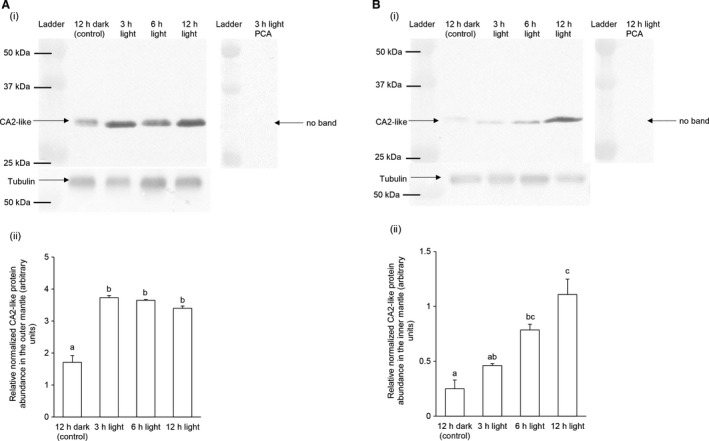
Effects of light on the protein abundances of carbonic anhydrase 2‐like (CA2‐like) in the outer mantle and inner mantle of *Tridacna squamosa*. The protein abundance of CA2‐like in (A) the outer mantle and (B) the inner mantle of *T. squamosa* kept in darkness for 12 h (control), or exposed to light for 3, 6, or 12 h. (i) Example of an immunoblot of CA2‐like and tubulin (left) and a peptide competition assay (PCA) was performed by preincubating the anti‐CA2‐like antibody with the immunizing peptide to check for antibody specificity (right). (ii) The optical density of the CA2‐like band for a 50 *μ*g protein load was normalized with respect to that of tubulin. Results represent means ± SEM (*N *=* *3). Means not sharing the same letter are significantly different from each other (*P *<* *0.05).

There were significant increases (~2‐fold) in the protein abundance of CA2‐like in the outer mantle of *T. squamosa* exposed to light for 3, 6, or 12 h as compared to the control (Fig. 5A; see Fig. S1A for the whole immunoblot). As for the inner mantle (Figs. 5B and S1B), a significant increase in the protein abundance of CA2‐like occurred after 6 or 12 h of exposure to light.

### Immunofluorescence microscopy

**Figure 6:**
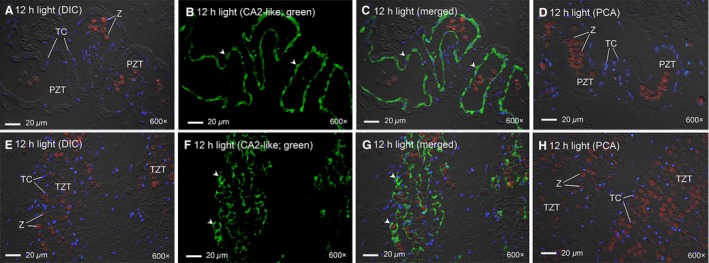
Immunofluorescence localization of carbonic anhydrase 2‐like (CA2‐like) in the outer mantle of *Tridacna squamosa*. Immunofluorescent localization of CA2‐like in the primary zooxanthellal tubes (PZT) and tertiary zooxanthellal tubes (TZT) of the outer mantle of *T. squamosa* exposed to 12 h of light (A–H). Autofluorescence from the zooxanthellae (Z) are shown in red with nuclei counterstained with DAPI in blue and overlaid with the respective differential interference contrast image (DIC) (A and E). Sections (A–D) show PZT located near the root of the outer mantle. PZT contains clusters of Z in one tubule. TZT (E–H) are localized at the inner fold of the outer mantle which faces sunlight (refer to Norton and Jones 1992 for an anatomical review). TZT encloses a single zooxanthella. Anti‐CA2‐like immunofluorescence staining is shown in green (B and F) and then overlaid with the respective DIC, red channel (autofluorescence from zooxanthellae) and DAPI staining (C and G). Peptide competition assay (PCA) was performed under the same experimental condition but with the anti‐CA2‐like antibody preincubated with the immunizing peptide in order to validate the antibody specificity and immunofluorescence (D and H). Arrowheads show cytoplasmic staining of CA2‐like in the host tubular epithelial cells (TC) surrounding PZT and TZT. Magnification: 600× for (A–H). Reproducible results were obtained from four individuals.

**Figure 7:**
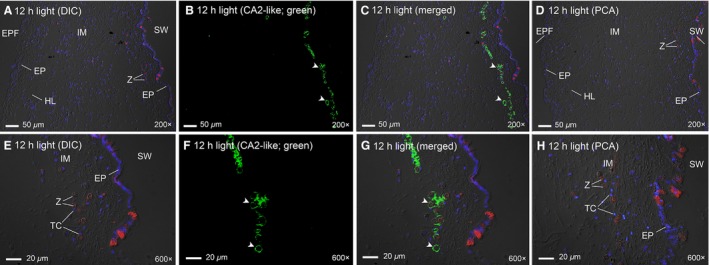
Immunofluorescence localization of carbonic anhydrase 2‐like (CA2‐like) in the inner mantle of *Tridacna squamosa*. Immunofluorescent localization of CA2‐like in the tertiary zooxanthellal tubes of the inner mantle (IM) of *T. squamosa* exposed to 12 h of light (A–H). Differential interference contrast image (DIC) is merged with red channel to show the localization of zooxanthellae (Z) and DAPI to show nuclei staining (A and E). Z are located near the seawater (SW)‐facing epithelium (EP) of the inner mantle (A–D). Tertiary tubules with Z are not found near the shell‐facing EP of the inner mantle adjacent to extrapallial fluid (EPF) (A–D). Anti‐CA2‐like immunofluorescence is shown in green (B and F) with nuclei counterstained with DAPI in blue and overlaid with the respective DIC (C and G). Peptide competition assay (PCA) was performed under the same experimental conditions but with anti‐CA2‐like antibody preincubated with the immunizing peptide in order to validate the antibody specificity and immunofluorescence (D and H). Arrowheads show CA2‐like staining of the cytoplasm in host tubular epithelial cells (TC) forming the tertiary zooxanthellal tubes. HL, hemolymph. Magnification: 200× for (A–D); 600× for (E–H). Reproducible results were obtained from four individuals.

CA2‐like was localized to the epithelial cells lining the primary (Fig. 6C) and tertiary tubules (Fig. 6G) surrounding the extracellular zooxanthellae in the outer mantle of *T. squamosa*. CA2‐like immunofluorescence was also detected in epithelial cells of the tertiary tubules located close to the seawater‐facing epithelium of the inner mantle (Fig. 7C and G), where the zooxanthellae would have a higher probability of exposure to light. Notably, the cells of the shell‐facing epithelium of the inner mantle lacked CA2‐like immunostaining (Fig. 7A–D).

## Discussion

### An overview of CAs and alga–invertebrate association

There is strong evidence to suggest that CAs play an important role in alga–invertebrate symbioses. Cnidarians containing endosymbiotic zooxanthellae have significantly higher CA activity (>29 times) than azooxanthellate species (Weis et al. 1989). With the administration of CA inhibitors (acetazolamide and ethoxyzolamide), photosynthetic rates in symbiotic corals and sea anemones are greatly reduced, denoting CA as a determining factor in photosynthetic productivity of zooxanthellae (Weis et al. 1989). In scleractinian corals, CAs may play a direct role in catalyzing ${\text{HCO}}_{3}^{-}$ formation within the subcalicoblastic space to enhance biocalcification (Tambutté et al. 2007). Indeed, a secretory CA has been cloned from the coral *Stylophora pistillata*, and this CA is localized to the calicodermis (Moya et al. 2008). For giant clams, two distinct CA isoforms with molecular masses of 32 kDa and 70 kDa have been identified from *T. gigas* (Baillie and Yellowlees 1998; Leggat et al. 2002, 2005), and the 70 kDa CA has been postulated to be involved in exogenous C_i_ uptake in the ctenidium. This is the first report of a CA2‐like of 29.6 kDa, with light‐dependent gene and protein expression levels, from the mantle tissue of *T. squamosa*. The cellular localization of CA2‐like indicates that it probably does not have a direct role in CO_2_/${\text{HCO}}_{3}^{-}$ metabolism during light‐enhanced calcification. Rather, it may participate in the supply of C_i_ to the extracellular zooxanthellae during photosynthesis.

### Molecular characterization of a host CA2‐like from *T. squamosa*

The active site of CA is located in a large conical cavity with a catalytic Zn^2+^ ion at its lowest position (Lindskog 1997). The Zn^2+^ ion is held in place by tetrahedral coordination with three conserved His residues and a water molecule/hydroxide ion (Christianson and Fierke 1996). These three His residues were conserved in the CA2‐like of *T. squamosa*. Overall, the residues critical for CA activity and characteristic of human CA2 were highly conserved in the CA2‐like of *T. squamosa*. When considered in conjunction with phenogramic analyses, it is logical to deduce that the CA2‐like of *T. squamosa* is an active cytosolic CA isoform with high CO_2_ hydration rates of a host origin.

All *α*‐CAs make use of a two‐step mechanism to catalyze CO_2_ hydration (Supuran 2008a). In the first stage, an OH^−^ bound to the Zn^2+^ at the active site reacts with CO_2_ to form a ${\text{HCO}}_{3}^{-}$. Almost immediately, a H_2_O molecule displaces the ${\text{HCO}}_{3}^{-}$ and attaches itself to the Zn^2+^ at the active site (Mikulski and Silverman 1804): ZnOH + CO_2_ ⇌ Zn${\text{HCO}}_{3}^{-}$ (+H_2_O) ⇌ ZnH_2_O + ${\text{HCO}}_{3}^{-}$. In the second stage, the Zn^2+^‐bound H_2_O molecule transfers one H^+^ to another residue within the active site of the enzyme, represented as X (Mikulski and Silverman 1804). This is the rate‐limiting step which regenerates the active enzyme with a Zn^2+^‐bound hydroxide for the next cycle of enzymatic reaction (Supuran 2008a): ZnH_2_O + X ⇌ ZnOH^−^ + XH^+^. This two‐stage reaction is vital for the catalytic efficiency of CAs and is made possible by its unique amphiphilic active site (Domsic and McKenna 1804). One half of the active site comprises a hydrophobic pocket which is responsible for binding to the nonpolar CO_2_ substrate (Merz 1991) and orienting it for attack by the highly nucleophilic Zn^2+^‐bound OH^−^ (Alterio et al. 2012). The identity of the amino acid residues that make up the hydrophobic substrate binding site has been elucidated as Val121, Val143, Leu198, Val207, and Trp209 in the CA2 of *H. sapiens* (Domsic and McKenna 1804). It has been reported previously that substitutions of Val121, Val143, and Leu198 with bulkier residues would reduce the volume of the CO_2_ binding cavity, thereby significantly decreasing CA activity (Fierke et al. 1991). All the residues of the hydrophobic cavity were conserved in the CA2‐like of *T. squamosa*.

The other half of the CA active site is lined with hydrophilic residues through which the polar hydration products (${\text{HCO}}_{3}^{-}$ and H^+^) are released toward the environment (De Simone et al. 2013). These residues also coordinate with H_2_O to form an ordered hydrogen‐bonded network (“proton wire”) which allows a proton to be transferred off the Zn^2+^‐bound water molecule to regenerate the active Zn^2+^‐hydroxide form of the enzyme (Tu et al. 1989; Fisher et al. 2007). Only four of the six residues known to form the hydrophilic portion of the active site in human CA2 were conserved in the CA2‐like of *T. squamosa*, of which, the His64 residue involved in the penultimate step of H^+^ transfer (Domsic and McKenna 1804) was conserved. It has been established that this particular residue is responsible for ensuring efficient H^+^ transfer and consequently the high catalytic rate of CO_2_ hydration in the CA2 of *H. sapiens* (Supuran 2008a). A mutation of His64 results in 20‐ to 30‐fold decrease in the enzymatic activity of CA2 (Tu et al. 1989).

### CA2‐like is localized to the cytoplasm of cells around the zooxanthellal tubules in the outer and inner mantle

Immunofluorescence microscopy demonstrated that CA2‐like was localized to the cytoplasm of host cells surrounding the zooxanthellal tubules in the outer and inner mantle of *T. squamosa*. Notably, the tertiary tubules and the associated zooxanthellae were located close to the seawater‐facing epithelium of the inner mantle whereby light is more readily available than the shell‐facing epithelium. The close association of the host CA2‐like with the symbiotic zooxanthellae in situ denotes that it may play an active role in supplying C_i_ to the zooxanthellae. On the other hand, the lack of immunofluorescence in the shell‐facing epithelium of the inner mantle indicates that CA2‐like may not participate directly in light‐enhanced calcification, and suggest the possible involvement of some other host CA isoforms yet to be identified. Of note, CA2 has been implicated in the maintenance of healthy bone structures in humans, not in terms of promoting calcification, but by providing H^+^ formed from CO_2_ hydration to dissolve and reabsorb the organic bone matrix (Supuran 2008b).

### Symbiotic zooxanthellae depend on host's transport systems for C_i_ supply

In alga–invertebrate association, even the combined metabolic CO_2_ of the host and symbionts are insufficient to meet the high C_i_ demands of photosynthesis (Hayes and Goreau 1977). While seawater contains a high concentration of dissolved C_i_, symbiotic zooxanthellae, unlike their free‐living counterparts, do not have direct access to this pool of exogenous C_i_ (Goiran et al. 1996). In symbiotic cnidarians, zooxanthellae are located intracellularly within the gastroderm layer and enclosed by a host‐derived symbiosome membrane (Roth et al. 1988). They are separated from the surrounding seawater by only two layers of host cells of 45–200 *μ*m thickness (Gattuso et al. 1999), making the diffusion path of C_i_ relatively straightforward. By contrast, *T. squamosa* is a much more complex organism with multiple organs that are spatially separated. The symbiotic zooxanthellae of giant clams reside mainly inside tertiary tubules located predominantly in the topmost 2 to 3 mm of the outer mantle (Norton and Jones 1992). Inside the outer mantle, the tertiary tubules are in close proximity with hemal sinuses which contain the hemolymph (Leggat et al. 2002). Leggat et al. (Leggat et al. 1999) reported that in light, hemolymph C_i_ fell from 1.22 to 0.75 mmol L^−1^, indicating that the hemolymph was the immediate source of C_i_ for the zooxanthellae during photosynthesis. Accordingly, the symbiotic zooxanthellae must rely on host transport mechanisms located in the membranes of the epithelial cells of the tubules to supply them with the C_i_ needed for photosynthesis.

### Light‐enhanced expression of *CA2‐like*/CA2‐like in the outer and inner mantle suggests its involvement in the increased supply of C_i_ from the host clam to symbiotic zooxanthellae

In photosynthetic organisms, ribulose‐1,5‐bisphosphate carboxylase‐oxygenase (RuBisCO) catalyzes a reaction essential to C_i_ fixation and requires CO_2_ as the obligatory substrate (Cooper et al. 1969). *Symbiodinium* harbors a unique form II RuBisCO (Rowan et al. 1996) which has very low affinity to CO_2_ but binds strongly to O_2_ (Morse et al. 1995). The kinetics of form II RuBisCO of *Symbiodinium* renders CO_2_ fixation ineffective (Reinfelder 2011) except at high *P*CO_2_. Indeed, CO_2_ has been reported as the preferred source of C_i_ for *Symbiodinium* isolated from giant clams (Yellowlees et al. 1993). Hence, the symbiotic zooxanthellae residing in the luminal fluid of the zooxanthellal tubules need to be supported with an increased supply of C_i_, as CO_2_, due to photosynthetic activities during insolation.

Our results suggest for the first time that CA2‐like may participate in the increased supply of C_i_ from the host clam to the symbiotic zooxanthellae through the epithelium of the zooxanthellal tubules because of the following reasons. First, the outer mantle exhibited the highest level of host *CA2‐like* gene expression which corroborates the highest density of tertiary tubules and zooxanthellae therein. Second, CA2‐like was localized to the epithelial cells that form the zooxanthellal tubules. Third, exposure to light induced significant increases in the protein abundance of CA2‐like in the outer mantle. In light, CO_2_ is rapidly hydrated to ${\text{HCO}}_{3}^{-}$ catalyzed by CA2‐like in the cytoplasm of the epithelial cells surrounding the zooxanthellal tubules. The ${\text{HCO}}_{3}^{-}$ generated intracellularly can be transported into the lumen of the zooxanthellal tubule via some kind of apical ${\text{HCO}}_{3}^{-}$ transporters (e.g., SLC4 family, Romero et al. 2013), which has yet to be identified. As CO_2_ is the preferred source of C_i_ for *Symbiodinium* isolated from giant clams (Yellowlees et al. 1993), ${\text{HCO}}_{3}^{-}$ must undergo hydration to form CO_2_ in the lumen of zooxanthellal tubules, and the hydration reaction requires a supply of H^+^. Indeed we have cloned the vacuolar‐type proton ATPase (VHA) subunit A, *ATP6V1A*, from the outer mantle of *T. squamosa*, and confirmed that its gene and protein expression levels can be enhanced by light (Y. K. Ip, unpubl. results). Furthermore, ATP6V1A was localized to the apical (lumen‐facing) membrane of the epithelial cells of the zooxanthellal tubules (Y. K. Ip, unpubl. results), indicating that VHA is positioned to secret H^+^ into the lumen of the tubules during light exposure. When taken together, these results suggest that the epithelial cells of the zooxanthellal tubules in *T. squamosa* may possess a light‐enhanced and host‐mediated carbon‐concentrating mechanism (CCM) to facilitate the supply of C_i_ to the symbionts.

### Perspectives

In response to poor RuBisCO enzyme kinetics, many marine phototrophs have evolved CCMs to increase their internal *P*CO_2_ to enhance catalytic efficiency (Giordano et al. 2005). These often involve intracellular CAs intrinsic to the phototrophs, which concentrate CO_2_ around RuBisCO to facilitate enzyme–substrate binding (Raven 1997). Hence, the CCMs need to be light‐dependent in order to effectively accumulate internal C_i_ to support photosynthesis (Badger et al. 1998), and indeed light‐stimulated CA activity has been reported for certain cyanobacteria and green algae (Palmqvist et al. 1994). Most CCMs described to date are from the phototroph, and there are indications that *Symbiodinium* living in association with giant clams may possess an algal CCM (Leggat et al. 1999). This is the first report on the possible presence of a host CCM in *T. squamosa* to benefit its symbionts. Efforts should be made in the future to examine the bicarbonate transporters involved in the host CCM of *T. squamosa*, and how the host CCM can work in synergy with the algal CCM to augment the supply of C_i_ from the host to the symbiont during photosynthesis.

## Conflict of Interest

None declared.

## Data Accessibility

## Supporting information




**Figure S1.** Effects of light on the protein abundances of carbonic anhydrase 2‐like (CA2‐like) in the outer mantle and inner mantle of *Tridacna squamosa*.
