# Supplementary figures and images for: Carbonic anhydrase 2‐like in the giant clam, Tridacna squamosa: characterization, localization, response to light, and possible role in the transport of inorganic carbon from the host to its symbionts
Source: Physiol Rep. 2017 Dec 4;5(23):e13494. doi: 10.14814/phy2.13494 (PMC5727267; doi:10.14814/phy2.13494)

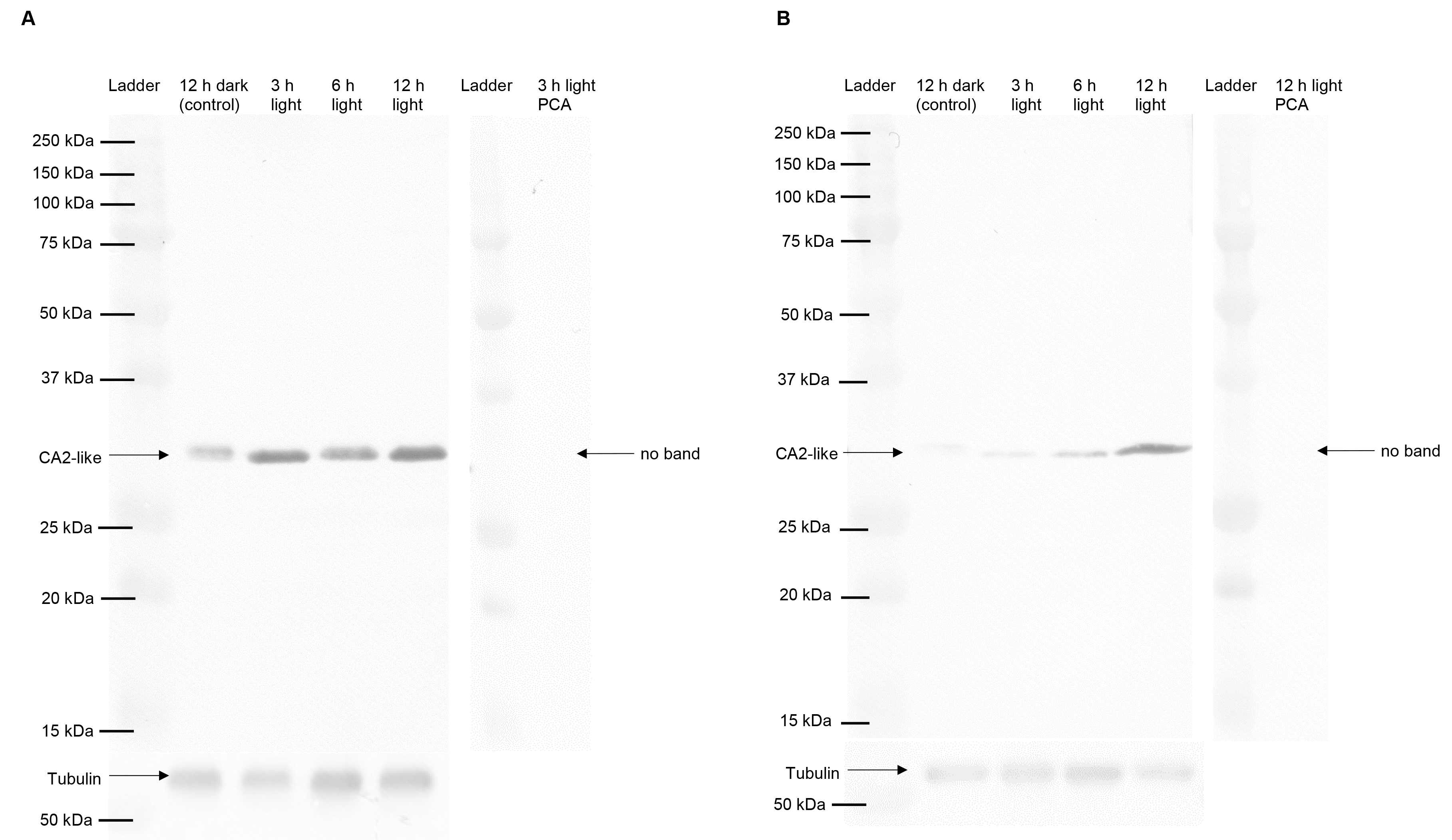

Supplement: Supplementary file 1 — Figure S1. Effects of light on the protein abundances of carbonic anhydrase 2‐like (CA2‐like) in the outer mantle and inner mantle of Tridacna squamosa. [file PHY2-5-e13494-s001.tif]
