# Supplementary material for: Carbonic anhydrase 2‐like in the giant clam, Tridacna squamosa: characterization, localization, response to light, and possible role in the transport of inorganic carbon from the host to its symbionts
Source: Physiol Rep. 2017 Dec 4;5(23):e13494. doi: 10.14814/phy2.13494 (PMC5727267; doi:10.14814/phy2.13494)
Supplement: Supplementary file 2 [file PHY2-5-e13494-s002.docx]

**FIGURE S1. Effects of light on the protein abundances of Carbonic Anhydrase 2-like (CA2-like) in the outer mantle and inner mantle of *Tridacna squamosa*.** The protein abundance of Carbonic Anhydrase 2-like (CA2-like) in the (**A**) outer mantle and (**B**) inner mantle of *Tridacna squamosa* kept in darkness for 12 h (control), or exposed to light for 3, 6 or 12 h. Examples of immunoblot of CA2-like, CA2-like with immunizing peptide and tubulin as the reference protein.
